# Effective Insensitiveness of Melamine Urea-Formaldehyde Resin via Interfacial Polymerization on Nitramine Explosives

**DOI:** 10.1186/s11671-018-2803-z

**Published:** 2018-12-10

**Authors:** Xinlei Jia, Jingyu Wang, Conghua Hou, Yingxin Tan, Yuanping Zhang

**Affiliations:** School of Environment and Safety Engineering, North University of China, Taiyuan, 030051 China

**Keywords:** MUF, Interfacial polymerization, GPBXs, Safety, Insensitiveness

## Abstract

To improve the safety of ammonium nitrate explosives, the melamine urea-formaldehyde resin (MUF resin) was selected for the preparation of three typical nitramine explosives (cyclotetramethylenetetranitramine, HMX; cryclo-trimethylenetrinitramine, RDX; and hexanitrohexaazaisowurtzitane, CL-20) based green polymer-bonded explosives (GPBXs) via interfacial polymerization. Meanwhile, the corresponding composite particles prepared by physical mixing and drying bath methods were studied and compared. The particle morphology, crystal structure, thermal stability, and safety performance of the resultant composite particles were characterized by scanning electron microscopy (SEM), powder X-ray diffraction (XRD), Fourier transform infrared (FT-IR) spectra, differential scanning calorimeter (DSC), and impact sensitivity test, respectively. SEM results showed that MUF was successfully coated on the surface of the three explosives, and different composite particles prepared by the same method have their own unique characteristics. Such effect is attributed to the resin’s ability to isolate and buffer external stimuli. It is obvious that the interfacial polymerization is an effective desensitization technique to prepare core-shell composite particles for explosives.

**Electronic supplementary material:**

The online version of this article (10.1186/s11671-018-2803-z) contains supplementary material, which is available to authorized users.

## Background

**Fig. 1:**
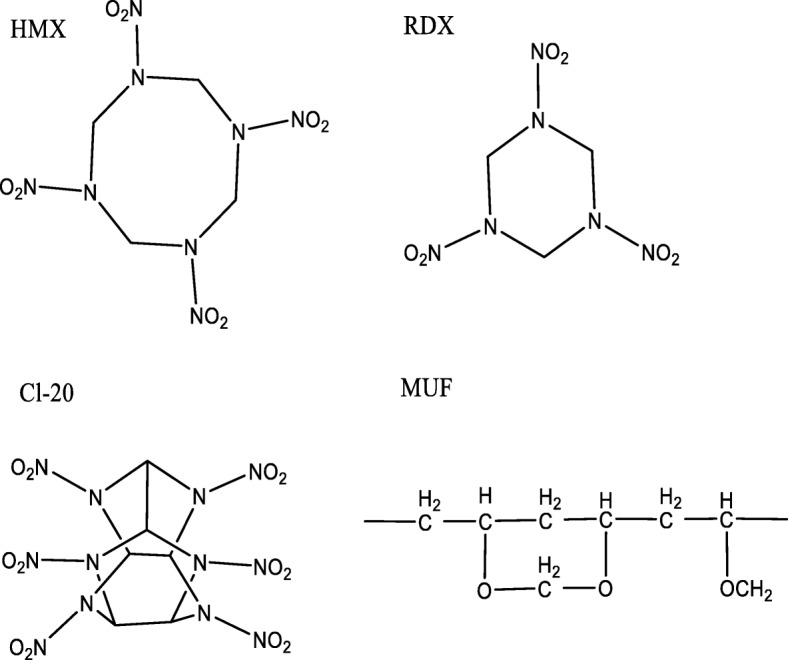
Molecular structure of RDX, HMX, CL-20, and MUF Binder. R-CH2OH is MF

As technology and weapon systems continue to evolve, ammunition is required not only to have high precision, high power, and long range for the weapon firepower system, but also to maintain relatively high safety in other environments. However, conventional explosives like hexahydro-1,3,5-trinitro-1,3,5-trizine (RDX), 1,3,5,7-teranitro-1,3,5,7-tetrazocane (HMX), and 2,4,6,8,10,12-hexanitro-2,4,6,8,10,12-hexaazaiso-wurtzitane (CL-20) are difficult to meet these requirements (molecular structures shown in Fig. 1), and the development of insensitive high explosives (IHEs) is considered as a desirable way to satisfy the application of weapon systems [1–3]. Many scholars at home and abroad are keen on the desensitization of nitramine explosives, usually using refinement [4, 5], coating [6, 7], and eutectic [8, 9] techniques to achieve the purpose of reducing sensitivity. The coating technology for energetic materials is a method of wrapping the modifier on the surface of the powder by a certain process to achieve the purpose of insensitivity, mainly including physical coating and chemical coating. Physical coating mainly refers to the formation of a certain coating layer on the surface of solid explosive particles by adsorption or external force. And common physical coating methods include water suspension method [10], crystallization coating method [11], spray-drying method [12], supercritical method [13], and phase separation method [14]. The chemical coating method refers to forming a coating layer on the surface of solid particles by metathesis, polymerization reaction, high-energy treatment, or the like in a certain medium. It is well known that the key indicators for the evaluation of a core-shell material are the degree of coverage, the mechanical strength and inhibition of self-nucleation for the coating shell [15]. Therefore, exploring novel coating techniques and finding new coating materials are effective ways to ensure that explosives possess a good core-shell structure and meet safety requirements. Our research is based on the two coating methods described above.

For decades, conventional “trialdehyde” binders have been the focus of research for scholars at home and abroad. With the comprehensive performance continuously improving, relevant reports on their applications have begun to appear in the field of energetic materials. In 2015, Yang et al. [16] used 3% melamine-formaldehyde (MF) resin monomer to coat CL-20, HMX, and RDX. The as-prepared particles had a compact and uniform MF resin coating layer, and their thermal stability and safety properties are greatly improved. This article has similarities with that report because we have also found that melamine-urea-formaldehyde resin is more suitable as an explosive binder material, compared with melamine-formaldehyde resin. MF resin has excellent performance; however, it is brittle and costly. The most important thing is that the MF resin product cannot be stored for a long time. If it is used as a coating material to fabricate energetic composite particles with a core-shell structure, it is likely to cause impaired properties of the particles. Li et al [17] prepared cyclotetramethylenetetranitramine at thermoplastic polyester-ether elastomer (HMX @ TPEE) energetic microspheres with particle size distribution ranging from 50 to 200 μm via an emulsion solvent evaporation (ESV) method, and the resultant particles had lower sensitivity and higher thermal stability. In this paper, an improved drying bath method was proposed, and the polymer-bonded explosives (PBXs) with excellent comprehensive performance were prepared by using melamine urea-formaldehyde resin (MUF resin) as the shell material and three different explosives as the core material. Wang et al. [18] obtained 30-nm cyclotetramethylenetetranitramine/nitrocellulose (HMX/NC) nanocomposites with good comprehensive property by an improved sol-gel-supercritical method. It can be seen that the choice of binder has a great influence on the morphology, particle size, and performance of the resultant PBXs.

As we all know, ultrasonic assistance has been widely applied to chemical synthesis and modification of functional materials [19, 20]. In our study, a green MUF binder with superior overall performance was prepared by a two-step synthesis method. Then, the as-prepared MUF binder was used as the shell material, and HMX, RDX and CL-20 as the core materials, respectively. First of all, using a simple physical mixing method, three different explosives/MUF particles (with MUF content of 5%) were fabricated via ultrasonic assistance. Subsequently, under the same conditions, the other six composite energetic particles were prepared by using improved drying bath method and an optimized interfacial polymerization method, respectively. In summary, for the first time, nine different composite energetic particles with the same MUF ratio were fabricated via ultrasonic assistance by the three different methods. Interestingly, through different methods, we obtained PBXs with different morphologies, such as apparent particle-exposed (physical mixing), irregular polygonal (drying bath method), and dense core-shell (interfacial polymerization) shapes. Surprisingly, as the esthetic appearance of the particle morphology increased, their thermal stability and safety performance improved. Through the research and analysis, the composite energetic particles prepared by interfacial polymerization are optimal in morphology, thermal stability, and safety performance. Therefore, in order to obtain composite energetic particles with the best comprehensive performance, it is preferred to consider interfacial polymerization method to prepare GPBX after determining the binder used.

## Methods

### Materials

HMX, RDX, and CL-20 were provided by Gansu Yinguang Chemical Industry Group Co. In our study, the raw materials were selected in drying bath process. When preparing explosive/MUF composites by physical mixing and interfacial polymerization methods, the raw materials were recrystallized according to the reference [21]. Dimethyl sulfoxide was obtained from Tianjin Fuchen Chemical Reagent Factory. Tween 80 and Span 80 were mixed as the composite emulsifier for explosives with M_Tween 80_: M_Span80_ of 0.57: 0.43. Triethanolamine (TEOA, used to adjust the pH value during the reaction) was from Tianjin Sailboat Chemical Reagent Technology Co., Ltd. Urea, formaldehyde, hydrochloric acid (5% dilute hydrochloric acid was used to adjust the pH value in the present study), and resorcinol (R-80) were provided by Tianjin Tianli Chemical Reagent Co., Ltd. Ammonium chloride was purchased from Tianjin Guangfu Technology Development Co., Ltd. Polyvinyl alcohol 2488 (PVA) was supplied by Qingdao Yousuo Chemical Technology Co., Ltd. Pure water was obtained from pure water supply of Taiyuan Iron and Steel Co., Ltd.

### Two-Step Synthesis of MUF Resin

The MUF binder with excellent comprehensive properties was prepared by a two-step process. Firstly, preparation of urea-formaldehyde resin prepolymer. 0.62 g of urea and 1.87 g of formaldehyde solution (the concentration is 37%) were mixed, and then the urea was sufficiently dissolved with a magnetic stirrer. The pH value of the mixture was tuned to 8.5~9.5 with triethanolamine. The solution was placed in a water bath at 65 °C and stirred for 1 h until a transparent and viscous urea-formaldehyde resin prepolymer was obtained. After cooling, HCl was added dropwise until the pH value of the solution was adjusted to about 3.5, and set it aside. Secondly, preparation of MUF. 1.87 g of prepolymer was added to 35 ml of deionized water to form an emulsion under uniform stirring. Subsequently, 8% PVA, 0.01 g of melamine, 0.125 g of resorcinol, and 0.06 g of ammonium chloride were successively added, and the pH value was adjusted to about 3.5 with dilute hydrochloric acid. Then the three-necked flask was placed in the water bath at 65 °C and reacted for 3~4 h, followed by standing, natural cooling and vacuum filtration. The solution was washed with deionized water, finally affording high-quality MUF resin. After drying, approximately 0.3 g of MUF was weighed.

### Preparation of Explosive/MUF Composite Particles by Interfacial Polymerization and Drying Bath Methods

The preparation of explosive/MUF composite particles by interfacial polymerization and drying bath methods is completely consistent with the preparation of urea-formaldehyde resin prepolymer in the two-step synthesis of MUF binder. However, the second step is obviously different.

**Fig. 2:**
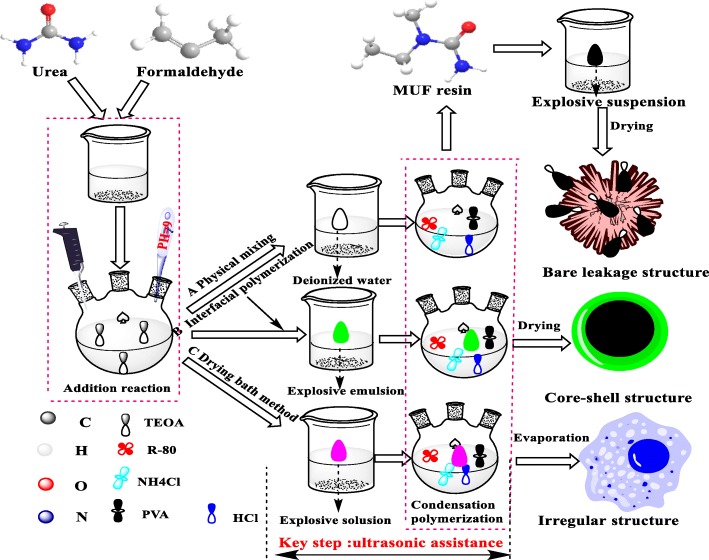
Schematic diagram of different methods for preparing HMX/MUF, RDX/MUF, and CL-20/MUF

In the fabrication of explosive/MUF composite particles by interfacial polymerization, 6 g of explosives was added to 35 ml of deionized water, and 0.01 g of span-80 was dropwise added as an emulsifier. Subsequently, the system was emulsified and sheared at a rate of 7000 rad/min for 30 min until a stable explosive emulsion was formed. The explosive emulsion replaced the deionized water in the two-step synthesis of MUF resin. The synthesis diagram is shown in B in Fig. 2 below.

In the preparation of explosive/MUF composite particles by the drying bath method, 6 g of explosive was dissolved in 35 ml of DMSO at 65 °C to form an explosive solution. The explosive solution replaced the deionized water in the two-step synthesis of MUF resin. After 3 to 4 h of reaction, a stable explosive/MUF milky mixture was formed. Then, the emulsion was placed in an oven and dried at 70 °C for 48 h, finally affording explosive/MUF composite particles. The synthesis diagram is shown in C in Fig. 2 below (drying bath). It should be noted that after the addition of the explosive emulsion, ultrasonic assistance must be performed in order to avoid the agglomeration of the binder and the explosive.

### Preparation of Explosive/MUF Composite Particles by Physical Mixing

In order to compare with the explosive/MUF particles prepared by the first two methods, we also prepared explosive/MUF particles by using a simple physical mixing method. The MUF binder, prepared by the two-step synthesis, was mixed with 6 g of explosive in 35 ml of deionized water, and then, the mixed solution was stirred in the water bath at 65 °C for 2 h. After that, the mixture was let to stand, followed by filtration and drying; explosive/MUF composite particles were obtained. The preparation sketch is shown in A in Fig. 2 (Physical mixing).

We labeled the samples prepared by interfacial polymerization method, drying bath method, and physical mixing method, as sample 1, sample 2, and sample 3, respectively.

### Characterization

Field-emission scanning electron microscopy (FESEM) images were taken on a MIRA3 LMH SEM (Tescan) at 10 k; X-ray diffraction (XRD) patterns were obtained using a DX-2700 (Dandong Haoyuan Corporation, Liao ning, China) X-ray diffractometer with Cu-Kα (40 kV, 30 mA) radiation at *λ* = 1.5418 Å. All samples were scanned from 5° to 50° with steps 0.03 and 6 s counting time; Fourier transform infrared (FT-IR) spectra were characterized by a Nicolet FT-IR 8700 Thermo (Waltham, MA, USA) with a wave number resolution of 4 cm^−1^ and a single average of 32 scans at number temperature; thermal analysis was performed on a differential scanning calorimeter (DSC-131, France Setaram Corporation, Shanghai, China) at heating rate of 10 °C/min. The drop hammer apparatus; the special height (*H*_50_) represents the height from which 2.500 ± 0.002 kg drop-hammer will result in an explosive event in 50% of the trials. In each determination, 25 drop tests were made to calculate the *H*_50_. And the mass of sample is 30 mg. The friction sensitivity of the samples was tested with a WM-1 friction instrument. In each determination, 25 samples were tested, and an explosion probability (*P*, %) was obtained. And the mass of sample is 20 mg. The particle size tested by QICPIC dynamic particle analyzer (SYMPATEC Co., Ltd., Germany), and its working environment is 5~35 °C; relative humidity is less than 85%; light source type is He-Ne laser; power is 2.0 mW; and wavelength is 0.6328 μm.

## Results and Discussion

### Morphology of the Samples

The morphology and structure of the raw RDX, HMX, and CL-20; the synthesized MUF binder (Additional file 1: Section S1); and the explosive/MUF composite particles prepared by the three methods were measured, respectively. The SEM image shows that the raw nitramine explosives exhibit polygonal in shape and uneven in size distribution. The appearance of the original MUF binder is spherical; however, it can be clearly seen that the particles are not full because its interior may be empty or partially water.

**Fig. 3:**
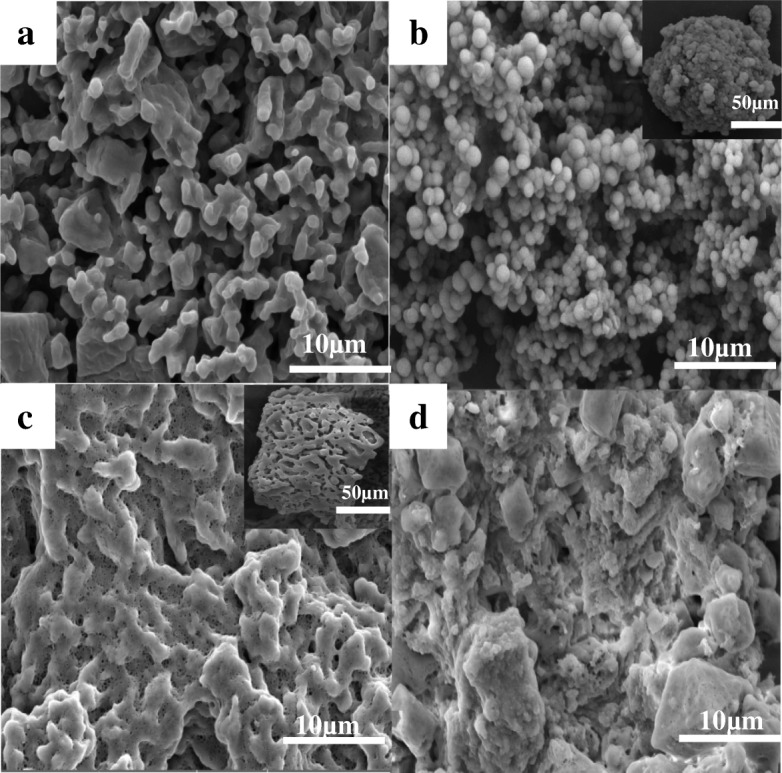
SEM images of the uncoated HMX (**a**), HMX/MUF-1 (**b**), HMX/MUF-2 (**c**), and HMX/MUF-3 (**d**); corresponding images with low magnification are inserted

**Fig. 4:**
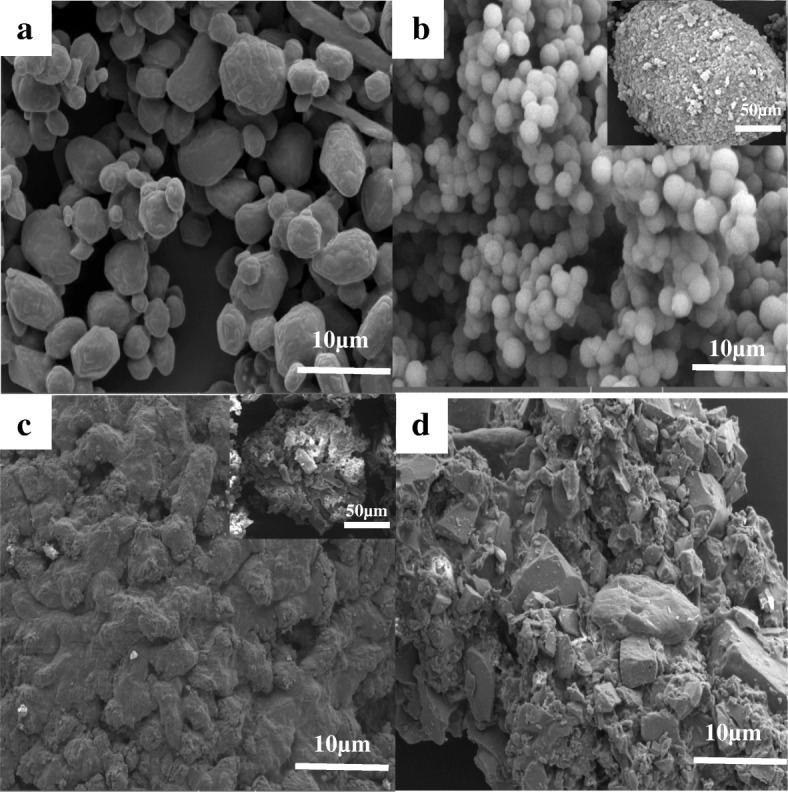
SEM images of the uncoated RDX (**a**), RDX/MUF-1 (**b**), RDX/MUF-2 (**c**), and RDX/MUF-3 (**d**); corresponding images with low magnification are inserted

**Fig. 5:**
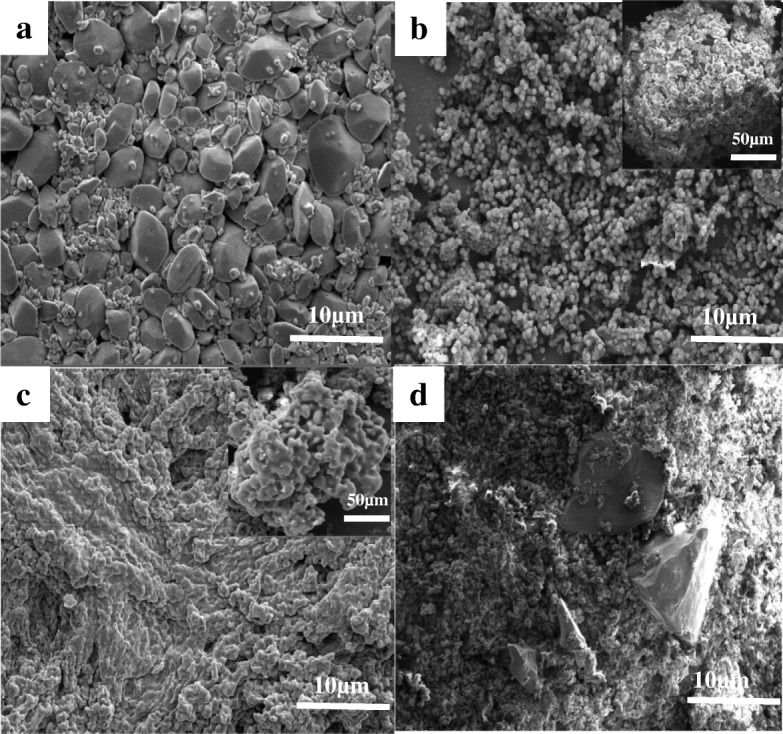
SEM images of the uncoated CL-20 (**a**), CL-20/MUF-1 (**b**), CL-20/MUF-2 (**c**), and CL-20/MUF-3 (**d**); corresponding images with low magnification are inserted

Compared with uncoated explosives (Figs. 3a, 4a, and 5a), the morphology of the explosive/MUF composite particles prepared by different methods is quite different, while the morphology of different explosive/MUF composite particles prepared by the same method has similar characteristics. The composite particles prepared by the physical mixing method have obvious particle exposure phenomenon, showing poor coating effect (Fig. 3d, 4d, and 5d). This is because it is difficult to distribute the binder evenly on the surface of the explosive only by mechanical action. The dispersion process of mechanical agitation alone is reversible. After the collision, the droplets will aggregate again, eventually achieving a dynamic balance that maintains a certain granularity. Uncontrollable mutual bonding occurs during the collision of droplets, which is beyond control.

The composite particles prepared by the drying bath method have distinctly dense coating layers. Surprisingly, after MUF completely encapsulates the explosive particles, it is difficult to form a complete sphere, most of which appears as dense but irregular particles (as shown in Figs. 3c, 4c, and 5c). And this phenomenon can be explained by the basic theory of interface chemistry [22]. During the solvent removal process, the viscosity of the explosive/MUF gradually increases as the solvent evaporates, and the dispersed particles tend to reaggregate together. On the other hand, since the solubility of the dispersant PVA in DMSO is small, when the MUF binders collide with each other, there is no good dispersing force, which causes them to stick to each other, eventually forming an irregular shape. In addition, the crystal growth theory [23] can also serve as a powerful support for explaining this irregular morphology. The drying bath method causes the explosive to undergo growth and development process of “the crystal embryo-nucleus-crystal”. Since the MUF system is in a metastable fluid phase and the system contains a variety of external surfaces, the dissolved explosive particles nucleate on these surfaces, which can reduce the nucleation barrier caused by the increase of surface energy of MUF and effectively decrease the surface energy barrier during nucleation of explosives. Explosive nucleation is preferentially formed at this unevenness, that is, non-uniform nucleation is also a cause of irregular particle morphology. From the illustration in Fig. 3d, we can see that the overall morphology of the HMX/MUF particles is “honeycomb”, which is related to the lower binding energy between MUF and HMX (Additional file 1: Section S2). As the evaporation proceeds, the MUF binder will gradually shrink. Too low binding energy between them renders MUF unable to completely encapsulate HMX, and there generates a strong internal stress, eventually forming the “honeycomb” shape [24].

The most interesting thing is that the explosive/MUF composite particles prepared by the interfacial polymerization method all possess a spheroidized structure, and the surface of the resultant particles is dense and smooth (as shown in Figs. 3b, 4b, and 5b). This is probably because the addition of the dispersant PVA decreases the surface tension of the water and improves the wettability, thus increasing the affinity between explosive molecules and the binder solution. The Hamaker constant is diminished simultaneously, and the attractive energy between particles is reduced, forming an effective steric hindrance. More important, the repulsive energy between the composite particles rises, which greatly enhances dispersibility between the explosive/MUF [25]. As depicted in the insert in Figs. 4b and 5b, numerous RDX/MUF and CL-20/MUF composite particles exhibit super solid spherical morphology, with their surfaces being dense and smooth. Surprisingly, the morphology of HMX/MUF particles shown in Fig. 3b is also spherical, but not as full as RDX/MUF and CL-20/MUF composite particles, attributing to the minimal binding energy between HMX and MUF. Too low binding energy makes the mixed system too stable, resulting in an obvious tendency for the MUF surface to shrink automatically. Therefore, although the HMX/MUF particles have a tendency to be spheroidized, they are not full.

### Crystal Structure of Samples

**Fig. 6:**
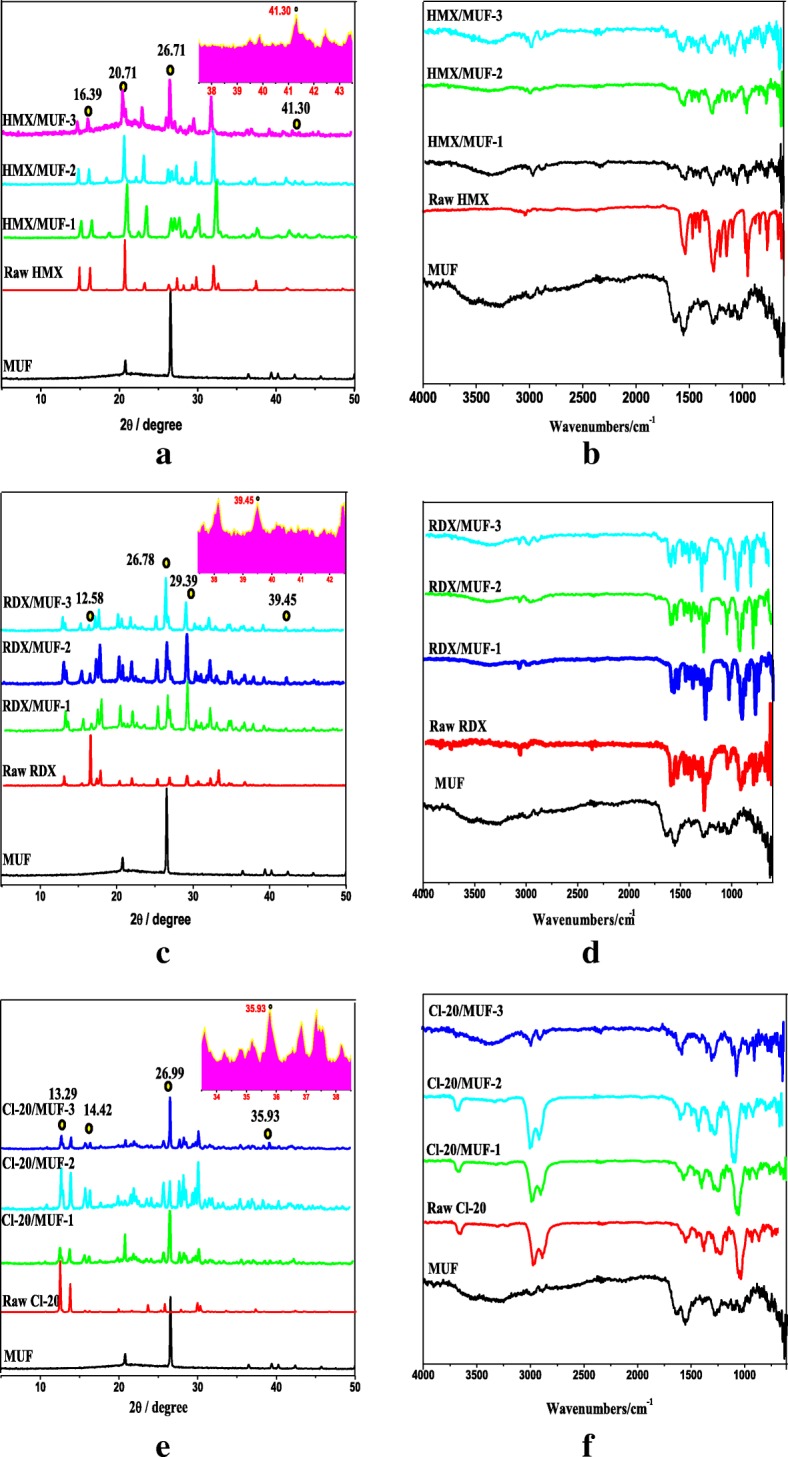
**a**–**f** XRD and FI-IR spectrum samples

To investigate whether the phase transformation of HMX and CL-20 occurred, XRD analysis is employed, and the results are shown in Fig. 6. Through analysis, it can be seen whether the crystal structure has changed during the preparation of the explosive/MUF composite particles. More importantly, X-ray diffraction analysis confirmed from the side that MUF was successfully coated on the surface of the explosive. From Fig. 6a, HMX/MUF-1, HMX/MUF-2, and HMX/MUF-3 contain almost all the diffraction peaks of the raw HMX. And similar phenomena also appear in the diffraction patterns of RDX and CL-20 composite particles, as shown in Fig. 6c, e. This indicates that the crystal structure of the explosive does not change during the whole preparation of the MUF/explosive by physical mixing, drying bath, and interfacial polymerization methods. Moreover, we have noticed a similar phenomenon among the three explosive/MUF composite particles, that is, the main diffraction peaks of the explosive/MUF composite particles are weakened and broadened as compared with the raw materials. For example, in the HMX/MUF, RDX/MUF, and CL-20/MUF diffraction patterns, the main diffraction peaks at 2*θ* = 16.39°, 12.58°, and 13.29° show the most obvious weakening and broadening phenomenon. This can be attributed to the “isotropic” physical properties of the amorphous MUF, resulting in an irregular arrangement for the resultant explosive/MUF particles in spatial distribution. Such periodic arrangement weakens the diffraction intensity of the explosive [26]. Most importantly, the diffraction peak of MUF is also present in the diffraction peak of the explosive/MUF composite particles. For example, in the HMX/MUF, RDX/MUF, and CL-20/MUF diffraction patterns, the diffraction peaks at 2*θ* = 26.71°, 26.78°, and 26.99° are much higher than the diffraction peak at the same position of the raw materials. Obviously, this is because the diffraction peak around 2*θ* = 27° is one of the most dominant diffraction characteristic peaks of MUF. Since the content of MUF accounts for only 5% of the explosive, the inconspicuous diffraction peaks present in MUF itself are less pronounced in the composite particles. As depicted in the magnified view of the diffraction peak inserted in each picture, compared with the original explosives, new diffraction peaks appear in the three explosive composite particles, such as at 2*θ* = 41.30° in the HMX sample diffraction pattern, 2*θ* = 39.45° in the RDX sample diffraction pattern and 2*θ* = 35.93° in the CL-20 sample diffraction pattern, which effectively confirms the existence of MUF binder in the explosive/MUF composite particles.

FI-IR analysis was performed to identify the molecular structure of the samples. On the whole, the composite particles prepared by the three different techniques contain almost all the stretching vibration peaks of the binder and the explosive. The measurement results confirmed from the side that the MUF resin successfully formed a protective layer on the surface of the explosive, corresponding to the XRD test results. It is well known that HMX has four different crystal structures, three pure crystal phases (α-HMX, β-HMX and δ-HMX), and one hydrate phase (γ-HMX). And β-HMX is generally considered as the most stable phase with high explosive energy, large density, and low sensitivity, which is of course related to the spatial structure of its monoclinic P21/c [27]. In the infrared spectrum of MUF, there is –C=O stretching vibration absorption peak at 1735 cm^−1^. While in the infrared spectrum of HMX, –NO_2_ and –CH_2_ stretching vibration absorption peaks appear near 1560 cm^−1^ and 2980 cm^−1^, respectively (as shown in Fig. 3b). It can be noticed that similar stretching vibration absorption peaks appear in the corresponding positions in the characteristic band of HMX/MUF, which means that the crystal structure of HMX will not be changed during the preparation via physical mixing, drying bath, and interfacial polymerization methods. Moreover, a similar situation is also found in the infrared spectrum of CL-20 particles (Fig. 6f), especially the stretching vibration peak of CL-20 particles in the fingerprint region 760 cm^−1^ demonstrates that Ɛ-CL-20 crystal structure did not change throughout the experiment [28].

### Thermal Properties

**Fig. 7:**
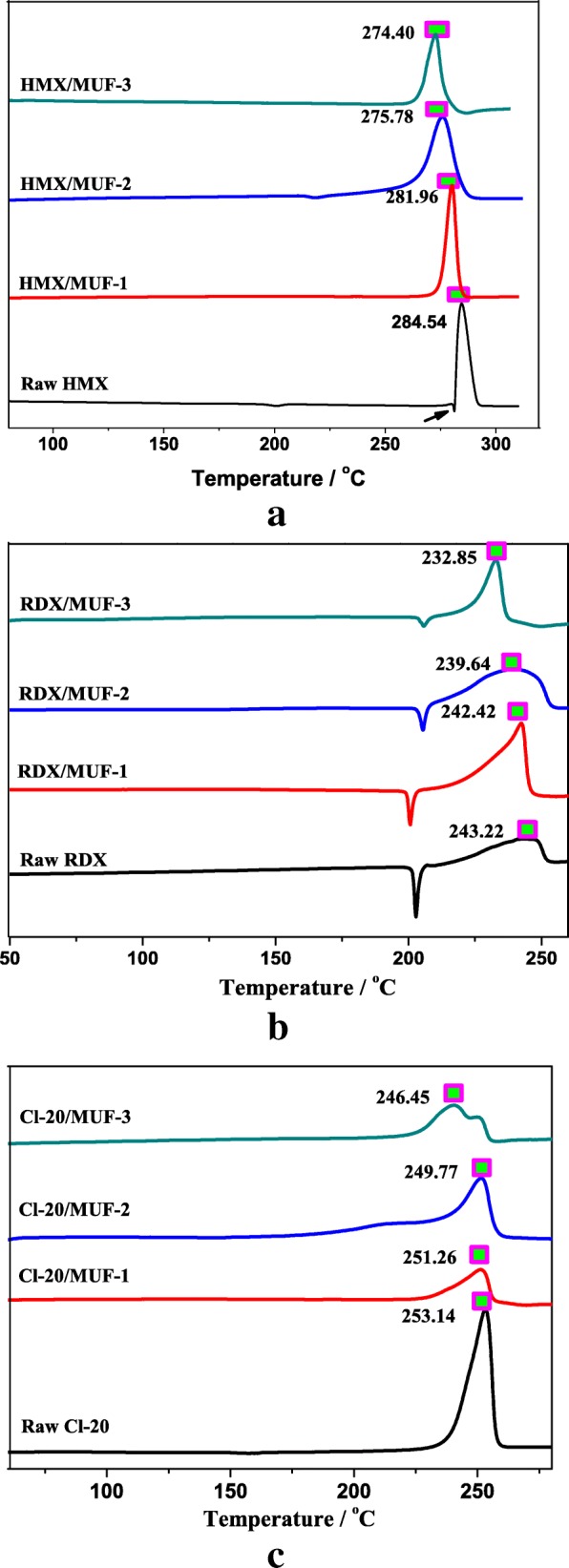
**a**–**c** DSC curves of samples collected at a heating rate of 10 °C/min

Probing the thermal decomposition process is very important for energetic materials [29]. In our research, DSC curves collected at a heating rate of 10 °C/min are obtained in Fig. 7. We have found some interesting phenomena about thermal decomposition of these three nitramine explosives. Overall, HMX and CL-20 have similar thermal decomposition characteristics (there is an endothermic peak of crystal transformation during thermal decomposition); however, the self-heating phenomenon of CL-20 is more serious than that of HMX. This is due to the fact that as a cage-type ammonium nitrate explosive, the cleavage of the molecular skeleton and the “heterogeneous condensed phase reaction” of the condensed phase exist simultaneously and exacerbate, while HMX is a type of “decomposition-melting” material, and its melting process is affected by the thermal decomposition process. In practice, HMX and RDX also have similar thermal behaviors, because both have the same branched chains. The difference is that HMX releases heat rapidly during thermal decomposition, and its DSC curve shows a steep and sharp peak (Fig. 7a). Because the decomposition of HMX is a heterogeneous process where the solid-liquid reaction proceeds simultaneously, whereas the decomposition of RDX is a homogeneous process in the molten state after the completion of melting. The accelerated reaction caused by the simultaneous phase change during the decomposition makes the decomposition of HMX more severe than that of RDX [30].

For HMX, the DSC curve shows an endothermic peak at 279.2 °C, subsequently an exothermic peak at 284.54 °C (Fig. 7a), attributing to the melting phase transition and the characteristic peak for the thermal decomposition of HMX, respectively [31]. Compared with raw HMX, the thermal decomposition temperatures of HMX/MUF-1, HMX/MUF-2, and HMX/MUF-3 all decreased. And the temperature of the composite particles prepared by interfacial polymerization, drying bath, and physical mixing methods reduced by 2.58 °C, 8.76 °C, and 10.14 °C, respectively. Similar results were reported as a lowering of decomposition temperature of HMX when it was coated with binder [32, 33]. Under the premise of containing 5% MUF, the decreasing degree is quite different for the decomposition peak temperatures of HMX-based composite particles fabricated by different methods; obviously, the effect of interfacial polymerization on the thermal decomposition performance of HMX is minimal. Similar situations can be seen in RDX/MUF and CL-20/MUF composite particles as well (as observed in Fig. 7c). This may be relevant to the coating morphology and compactness of HMX/MUF, and the uniform coating contributes to the stability of the thermal decomposition process of the composite particles. Therefore, in order to improve the thermal stability of the composite particles, it is an effective means to select a coating material with excellent thermal properties. In addition, under the premise of choosing a particular binder, it may be a good way to consider using interfacial polymerization method to prepare composite particles.

### Sensitivities

**Fig. 8:**
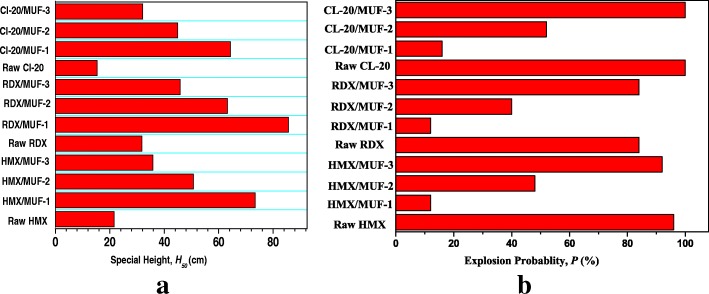
Impact sensitivity of samples: **a** impact sensitivity and **b** friction sensitivity

**Fig. 9:**
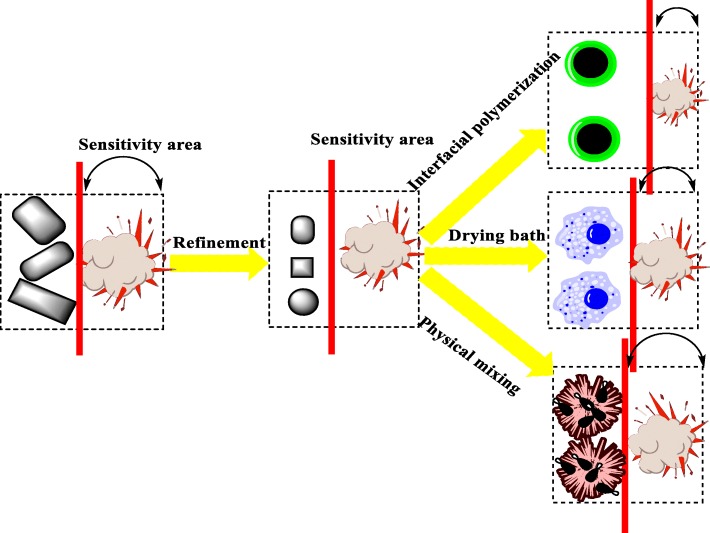
Schematic diagram of sensitivity

To investigate the safety performance of the samples, tests of the impact and friction sensitivities were performed, and the results are presented in Fig. 8. As we can see, among the desensitization treatments performed on HMX, RDX, and CL-20, MUF has the most significant desensitizing effect on composite particles prepared by the interfacial polymerization method. Compared with raw HMX, RDX and CL-20, the characteristic height *H*_50_ increased from 21.6 cm, 31.8 cm, and 15.3 cm to 73.4 cm, 85.6 cm, and 64.03 cm, respectively (Fig. 8a), thus significantly improving the safety performance. Besides, it can be seen from Fig. 8b that the friction sensitivity of GPBX fabricated by these three different methods is lower than that of uncoated explosive compounds. Interestingly, the three samples prepared by the interfacial polymerization exhibit the lowest friction sensitivity. More importantly, compared to previous reports [7, 18, 26], the safety performance of GPBX fabricated by interfacial polymerization is optimal. The desensitization effect is amazing. This can be explained by the hotspot theory [34]. MUF is successfully coated on the surface of HMX, which can produce a certain buffer effect under external mechanical stimulus, effectively slowing down the formation of hot spots. Schematic diagram of desensitization effect of composite particles prepared by three different techniques can be seen from Fig. 9. Obviously, with the same proportion of MUF binder, the composite particles fabricated by interfacial polymerization possess the most distinct desensitization effect, attributing to more uniform particle morphology. The uniform, small particle size distribution between the particles increases the gap between themselves, and the force area of the same quality composite particles increases, which reduces the stress concentration between the particles and effectively prevents the formation of local hot spots.

## Conclusions

Via ultrasonic assistance, nine different composite particles were fabricated by a simple physical mixing method, an improved drying bath method, and an optimized interfacial polymerization method. XRD and FT-IR analysis did not show any change in the crystal structure before and after the preparation of HMX and CL-20, still maintaining β-HMX and Ɛ-CL-20, respectively. Compared with the raw explosives, the thermal decomposition peak temperature of the composite energetic particles after adding MUF was reduced; however, the reduction effect of the thermal decomposition peak temperature of the sample 3 was not significant. The characteristic height H_50_ of the composite particles prepared by interfacial polymerization method increased by three to four times, most obviously improving the safety performance. In short, HMX/MUF, RDX/MUF, and CL-20/MUF particles prepared by each method have similarities in morphology, particle size, and even performance. In particular, the three composite particles fabricated by interfacial polymerization method possess better thermal stability and safety performance with smooth surfaces, dense and uniform coating layers. Therefore, in order to improve the thermal stability of the composite particles, it is an effective approach to select a coating material with excellent thermal performance. And under the premise of choosing a specific binder, it may be effective to prioritize the use of interfacial polymerization method to prepare composite particles. This study provides certain reference for the application of high-energy and low-sensitivity ammunition in weapon firepower and rocket systems.

## Additional File

Additional file 1: Supporting information for preparation of HMX, RDX, and CL-20 based GPBX via ultrasonic assistance with reduced sensitivity. (DOCX 3044 kb)

## Abbreviations

CL-20: Hexanitrohexaazaisowurtzitane
DSC: Differential scanning calorimetry
ESV: Emulsion solvent evaporation
FI-IR: Fourier-transform infrared spectra
GPBX: Green polymer-bonded explosives
HMX: Cyclotetramethylenetetranitramine
IHEs: Insensitive high explosives
MF: Melamine formaldehyde
MUF: Melamine-modified urea-formaldehyde
NC: Nitrocellulose
PF: Phenolic resin
RDX: Cyclotrimethylenetrinitramine
SEM: Scanning electron microscopy
UF: Urea formaldehyde
XRD: X-ray diffraction
