# Supplementary material for: Effective Insensitiveness of Melamine Urea-Formaldehyde Resin via Interfacial Polymerization on Nitramine Explosives
Source: Nanoscale Res Lett. 2018 Dec 10;13:402. doi: 10.1186/s11671-018-2803-z (PMC6288043; doi:10.1186/s11671-018-2803-z)
Supplement: Supplementary file 1 — Supporting information for preparation of HMX, RDX, and CL-20 based GPBX via ultrasonic assistance with reduced sensitivity. (DOCX 3044 kb) [file 11671_2018_2803_MOESM1_ESM.docx]

**Section S1**


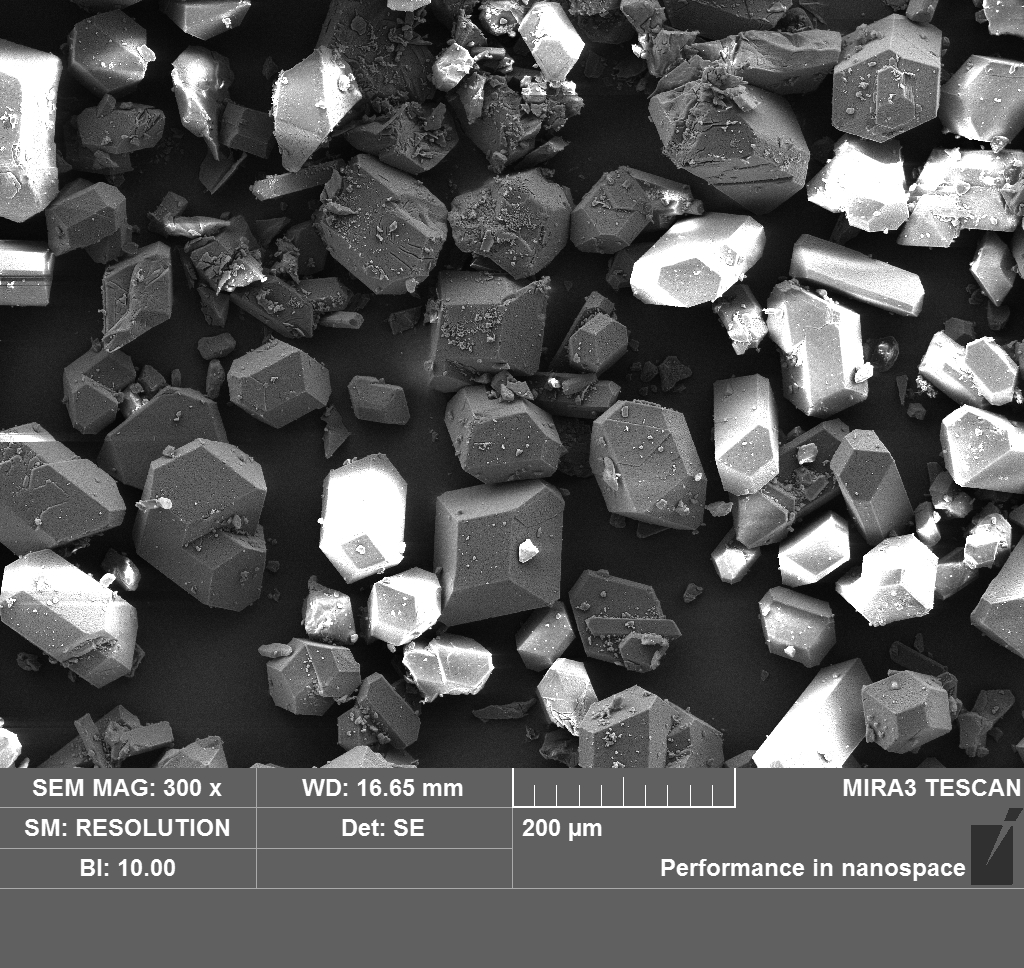

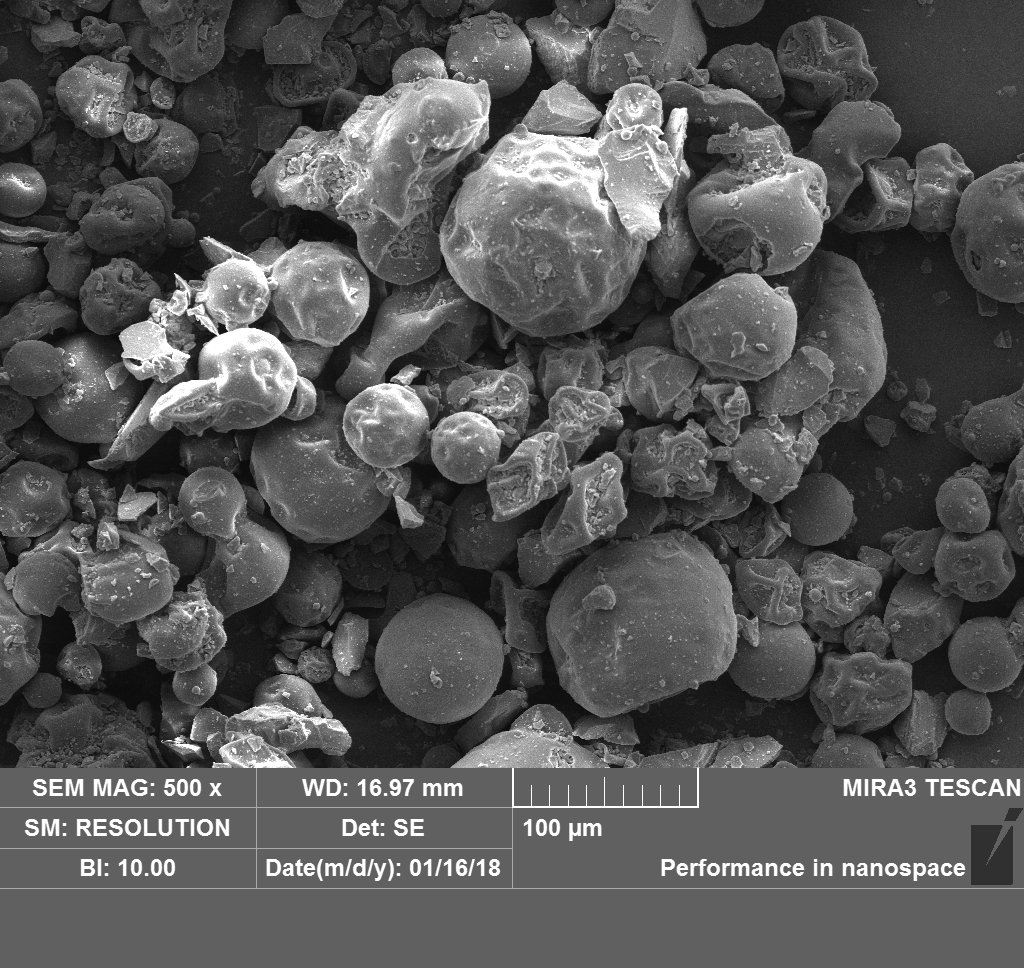


b

a


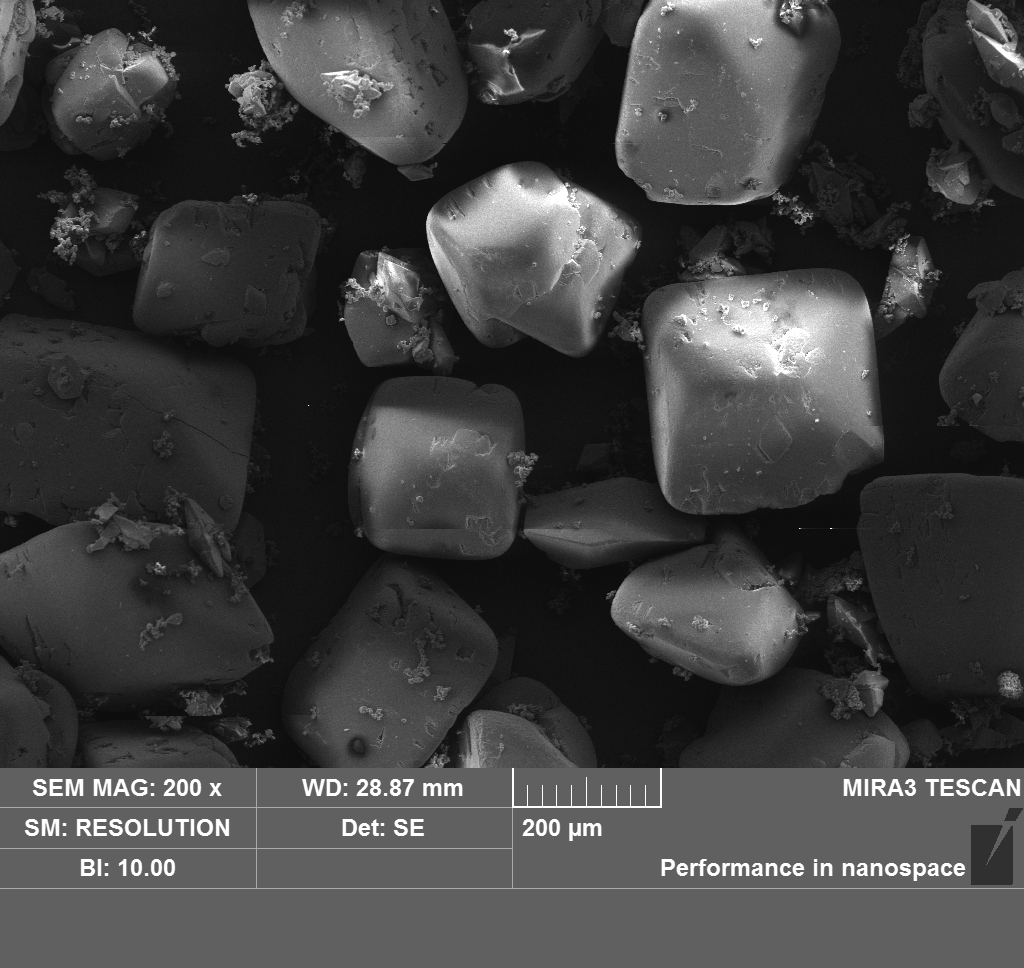

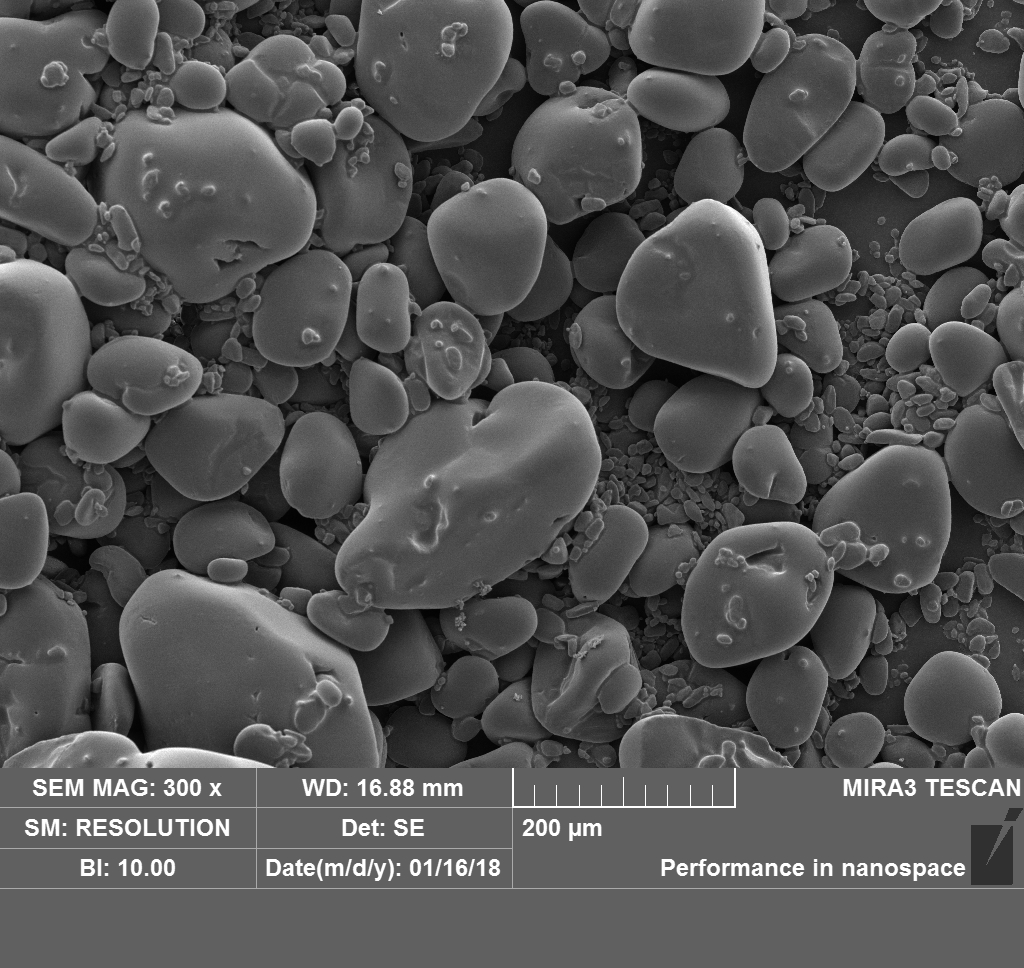


d

c

**Fig.S1** SEM images of raw materials: (a) raw MUF binder, (b) raw HMX, (c) raw RDX, (d) raw CL-20

**Section S2**

Molecular dynamics (MD) method was used to simulate the structure and properties of HMX/MUF, RDX/MUF and CL-20/MUF, respectively. The Amorphous Cell module in Material Studio was used to geometrically optimize the model in the pcff force field. After reaching equilibrium, 3% MUF binder was placed on the sliced explosive crystal surface. Building a periodic structure, MD simulation was performed by using Forcite module in pcf force field[1,2].

MD processing details are shown in the table below.

**Tab.S1** MD simulation process parameters

| Forcefield | Non-bond | Summation method | Ensemble | Simulation temperature |
| --- | --- | --- | --- | --- |
| pcff | vdW,Coulomb | Atom based,Ewald | NVT | 298K |
| Cut-off distance | Time step | Frame output | Thermostat | Energy diviation |
| 15.5Å | 1 fs | every 5000 steps | Anderson | 50000 kcal/mol |
| Total simulation time | Buff width | Number of steps | Initial velocities | Quality |
| 10ps | 0.5Å | 10000 | Random | Fine |

Unit cell model diagrams of the complexes in the MD simulation are shown below:


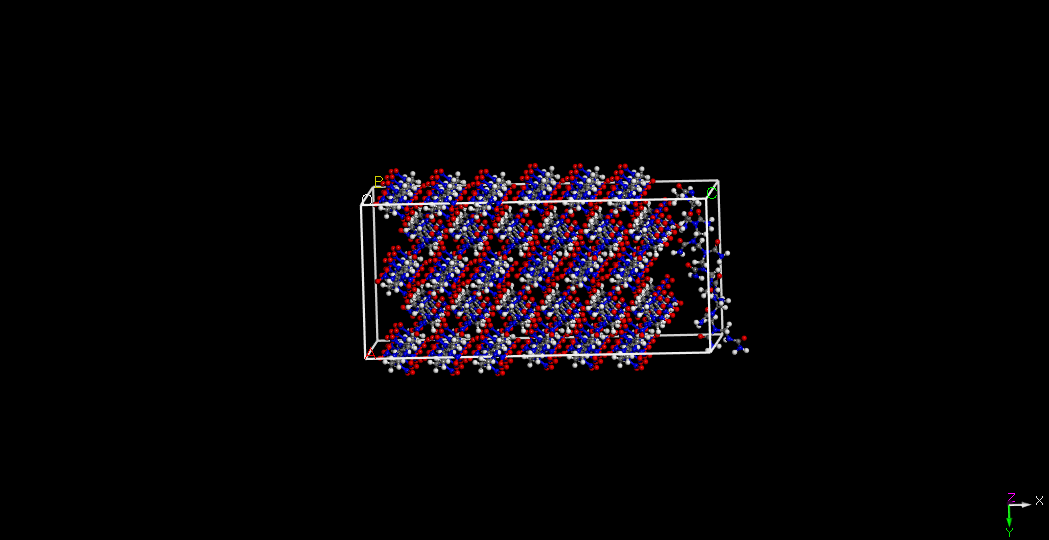


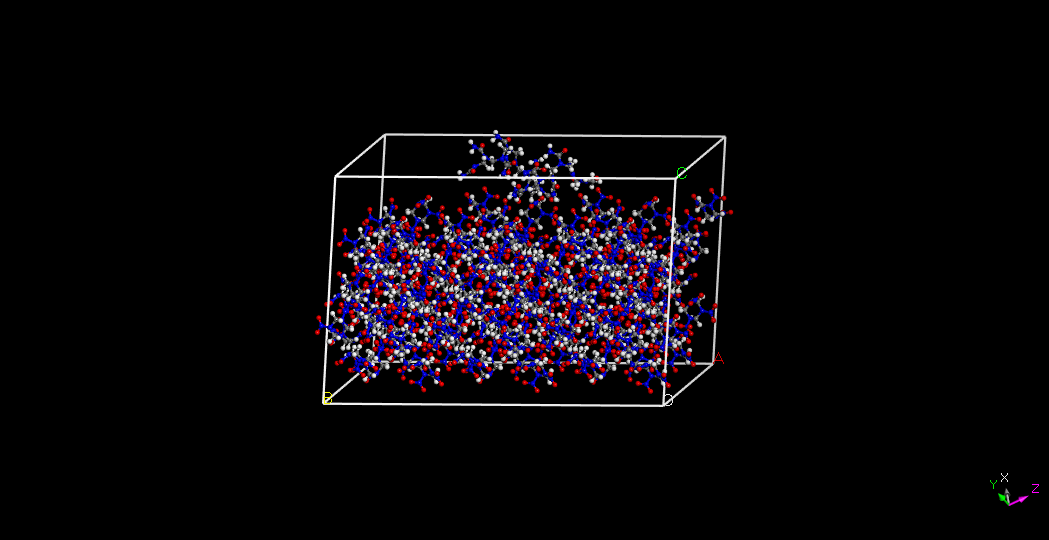

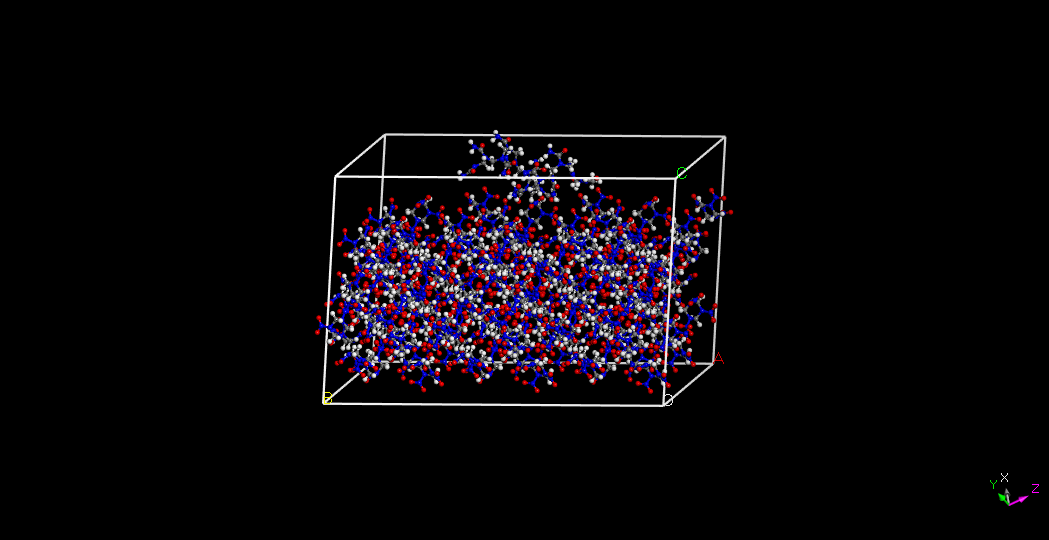


(a)

(c)


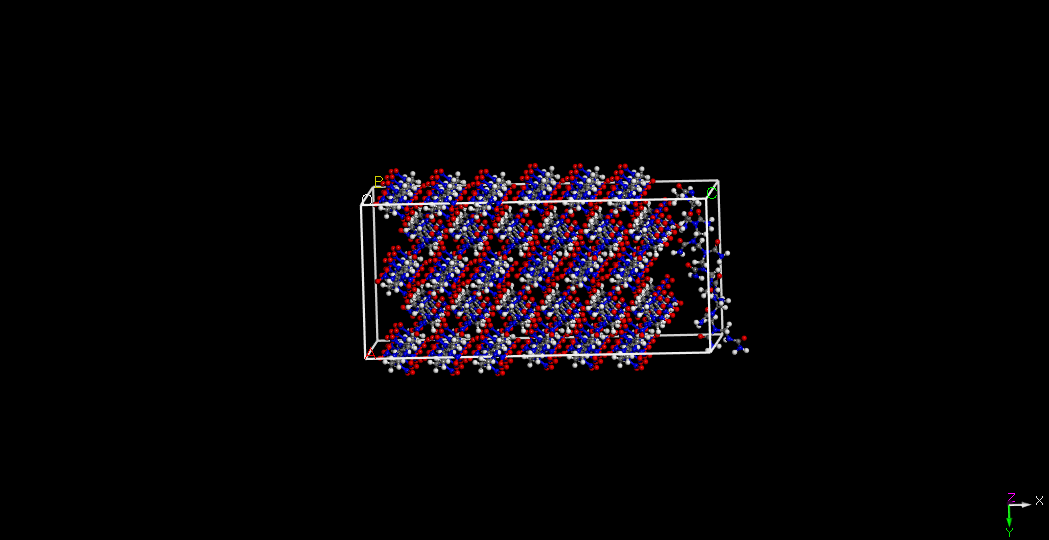


(b)

(b)

**Fig.S2** (a) HMX/MUF unit cell model (b) RDX/MUF unit cell model (c) CL-20/MUF unit cell model

The curves of energy and temperature with time in MD simulation is shown below:

**Fig. S3** The curves of energy and temperature of composite particle system with time (a) HMX/MUF (b) RDX/MUF (c) CL-20/MUF

The binding energy E_bind_ of PBX system is an important index for evaluating the compatibility of HMX and polymer. The formula is as follows:

 （1）

Where E_PBX_ is the average total energy of PBX; E_HMX_ and E_poly_ are the average single point energy of HMX and polymer, respectively. The binding energy of PBX can be obtained through simulation, listed in TableS2.

**Tab.S2** The binding energy of PBX system

| PBX system | HMX/MUF system | RDX/MUF system | CL-20/MUF system |
| --- | --- | --- | --- |
| Binding energy/kcal·mol^-1^ | 34.22 | 138.24 | 378.32 |

References

[1] Sun H，Mumby SJ，Maple JR，Hagler AT. An ab Initio CFF93 All-Atom Force Field for polycarbonates[J]. J,Am.Chem.Soc.，1994，116（7）：2978-2987.

[2] Andersen H. C. Molecular dynamics simulation at constant pressure and/or temperature [J]. J. Chem. Phys., 1980, 72: 2384-2395.
